# Advanced Computer Vision Approaches in Biomedical Image Analysis

**DOI:** 10.1155/2014/347265

**Published:** 2014-07-03

**Authors:** Anke Meyer-Baese, Claudia Plant, Juan Manuel Gorriz Saez

**Affiliations:** ^1^Department of Scientific Computing, Florida State University, Tallahassee, FL 32306-4120, USA; ^2^Helmholtz Center Munich, Technical University of Munich, 80333 Munich, Germany; ^3^Department of Signal Theory, Networking and Communications, University of Granada, 18071 Granada, Spain

Medical imaging is today becoming one of the most important visualization and interpretation methods in biology and medicine. The past decade has witnessed a tremendous development of new, powerful instruments for detecting, storing, transmitting, analyzing, and displaying images. These instruments are greatly amplifying the ability of biochemists, biologists, medical scientists, and physicians to see their objects of study and to obtain quantitative measurements to support scientific hypotheses and medical diagnoses. Noise, artifacts, and weak contrast are the cause of a decrease in image quality and make the interpretation of medical images very difficult. These sources of interference, which are of a different nature for mammograms than for ultrasound images, are responsible for the fact that conventional or traditional analysis and detection algorithms are not always successful. The biomedical imaging scene is one of the most challenging since we have to deal not only with non-Gaussian, nonstationary, and nonlinear processes (transients, bursts, and ruptures) but also with mixtures of components interacting in a quite complicated form. Therefore, much of the research done today is geared towards improvement of the reduced quality of the available biomedical imaging material.

The aim of this special issue is to present the current state-of-the-art in the theory of advanced computer vision approaches and applications to biomedical image analysis and modeling. In this special issue, we have invited articles that explore novel problems in biomedical imaging that require advanced computer vision approaches. Three papers address the important aspect of segmentation in medical images, two papers deal with automated detection, and three others propose theoretical and computational or hardware solutions for encountered artifacts.

One of the papers evaluates the feasibility of computer aided malignant tumor detection using the traditional texture analysis applied on two-compartment-based parameter pseudoimages of dynamic contrast enhanced magnetic resonance (DCE-MR) breast image data. The innovation lies in the through-plane assessment capability. The paper shows that neural network classification using the textural parameters for automated screening of two-compartment-based parameter pseudoimages of DCE-MRI as input data represents a useful tool for the radiologists in the preassessment stage by revealing possible cancerous regions and in the postassessment stage by reviewing the segmentation especially in analyzing complex DCE-MRI cases.

A paper identifies and analyzes the artifacts produced by the simultaneous use of two probes of different or equal operating frequencies. These artifacts arising in 3D ultrasound images are of obscure nature and represent a challenging task in the daily clinical environment.

Another paper addresses the important problem for automated segmentation of medical images in the presence of intensity inhomogeneity and proposes a novel kernel-based fuzzy level set method to alleviate this problem and achieves in practical examples a high accuracy and is invariant to the inhomogeneity degree.

A paper proposes a novel method based on histogram equalization as a preprocessing step to improve the convergence rate in an affine registration algorithm. Its applicability is demonstrated on SPECT and PET brain images resulting from several neurodegenerative diseases.

One of the papers presents a new image segmentation method based on multiple active contours guided by differential evolution, called MACDE. The segmentation method uses differential evolution over a polar coordinate system to increase the exploration and exploitation capabilities regarding the classical active contour model. The authors successfully demonstrated that MACDE outperforms the classical active contour model and the interactive Tseng method in terms of efficiency and robustness for obtaining the optimal control points and attains high accuracy segmentation.

Another paper introduces a novel segmentation technique using 3D statistical features extracted from the volume image and is based on unsupervised vector quantization and fuzzy clustering techniques without using any a priori information. The resulting fuzzy segmentation method addresses the problem of partial volume effect (PVE) and has been assessed using real brain images from the Internet Brain Image Repository (IBSR).

A paper describes an algorithm for the automatic detection of white blood cells embedded into complicated and cluttered smear images. The technique, which is based on the differential evolution (DE) algorithm, transforms the detection task into an optimization problem whose individuals represent candidate ellipses. An objective function evaluates if such candidate ellipses are actually present in the edge map of the smear image. In experimental studies, white blood cell images with a varying range of complexity are included to validate the efficiency of the proposed technique in terms of its accuracy and robustness.

One of the papers is more visionary and presents several low-cost hardware implementation approaches for the new generation of tablets and/or smartphones for estimating motion compensation and segmentation in medical images. These systems have been optimized for breast cancer diagnosis using magnetic resonance imaging technology with several advantages over traditional X-ray mammography and also address the challenge of offering a medical tool that runs on widespread portable devices to aid in patient diagnostics.

*Anke Meyer-Baese* *Anke Meyer-Baese* *Claudia Plant* *Claudia Plant* *Juan Manuel Gorriz Saez* *Juan Manuel Gorriz Saez*

